# Supplementary material for: Pre-analytical variables affecting breast cancer biomarker expression: A controlled single-specimen study of fixation duration, cold ischemia time, and fixative preparation in a low-resource setting
Source: PLoS One. 2026 Jun 5;21(6):e0343185. doi: 10.1371/journal.pone.0343185 (PMC13240924; doi:10.1371/journal.pone.0343185)
Supplement: Attachment — Submitted filename: Response_to_Reviewers_PONE-D-26-05253.docx [file pone.0343185.s003.docx]

**Response to Reviewers**

**Manuscript PONE-D-26-05253**

*Pre-analytical variables affecting breast cancer biomarker expression: a controlled single-specimen study of fixation duration, cold ischemia time, and fixative preparation in a low-resource setting*

Dear Dr. Kaminski,

We thank you and the three reviewers for the careful and constructive evaluation of our manuscript. The comments have helped us considerably strengthen the work, particularly in terms of framing, interpretation, and transparent discussion of the study's limitations. We are grateful for the opportunity to submit a revised version.

In this revision, we have:

• Revised the title of the manuscript to reflect the three pre-analytical variables actually investigated (fixation duration, cold ischemia time, and fixative preparation) and to make the exploratory, single-specimen nature of the study explicit from the first line;

• Re-framed the entire manuscript as an exploratory, proof-of-concept, single-specimen experiment throughout the abstract, introduction, results, discussion, and conclusion;

• Added explicit statistical analyses for the effect of fixation duration (Spearman correlations and one-way ANOVA across fixation-duration windows), which were previously missing;

• Added a new Discussion subsection dedicated to the Effect of Fixation Duration, complementing the existing subsections on fixative preparation and cold ischemia time, so that each of the three pre-analytical variables is now discussed in its own dedicated subsection;

• Acknowledged biological heterogeneity (intra-tumoral and inter-tumoral) and explicitly stated that tumors with lower or heterogeneous baseline biomarker expression may be more vulnerable to pre-analytical variation than our index case;

• Clarified that the novelty of the work lies in the combined, controlled, factorial evaluation of the three pre-analytical variables, not in any of the individual effects;

• Grounded the choice of extended fixation durations (up to 144 h) in our own prior empirical observations at the same institution, published as Ndengue et al. (Journal of Science and Diseases, 2025), with explicit distinction between biopsies (up to 83 h), nodulectomies (up to 82 h), and mastectomies (up to 147 h);

• Fully restructured and renumbered the reference list in strict order of first appearance in the text, as required by PLOS ONE Vancouver style; updated to cite the current ASCO/CAP guidelines (Allison et al. 2020 for ER/PR; Wolff et al. 2023 for HER2); and added recent 2024 literature on cold ischemia time in breast cancer (Shi, Ghlichloo, Fadare 2024);

• Tempered the language throughout to distinguish statistical from clinical relevance and to avoid premature extrapolation to resource-limited clinical practice.

Below we address every point raised by the editor and each reviewer. Reviewer comments are shown in italics on a light-grey background; our responses and the corresponding manuscript changes follow. Page and line numbers refer to the revised clean manuscript ("Manuscript" file) unless otherwise stated. In the tracked-changes version of the manuscript, insertions are shown in blue and deletions in red with strikethrough; a note to this effect is placed at the end of that file.

# **Preliminary note: change of manuscript title**

Following careful consideration of the editor's call for improved framing and of Reviewer #2's observation that statistical testing of the effect of fixation duration was missing, we have revised the title of the manuscript. The previous title mentioned only two of the three pre-analytical variables actually manipulated in the study and did not convey the exploratory, single-specimen nature of the design.

The previous title was:

*"Effect of Cold Ischemia Time and Fixative Preparation on Breast Cancer Biomarker Expression: Implications for Resource-Limited Settings"*

The revised title is:

*"Pre-analytical variables affecting breast cancer biomarker expression: a controlled single-specimen study of fixation duration, cold ischemia time, and fixative preparation in a low-resource setting"*

The revised running title is: "Fixation, ischemia and breast biomarkers" (40 characters, within the PLOS ONE short-title limit).

This change makes the title faithful to the three-factor factorial design, explicit about the exploratory nature of the work, and consistent with the re-framing requested by the reviewers throughout the manuscript.

# **Preliminary note: restructuring of the reference list**

The reference list has been entirely restructured in this revision. The reasons and the procedure are as follows:

• Three cited guideline references were superseded versions and have been replaced by their current versions: Hammond et al. 2010 → Allison et al. 2020 (ASCO/CAP ER/PR update); Wolff et al. 2013 and Wolff et al. 2018 → Wolff et al. 2023 (ASCO-CAP HER2 update).

• Two new references have been added: Ndengue et al. 2025 (our prior retrospective study at the same institution, which documents fixation-duration distributions and motivates the range tested here) and Shi, Ghlichloo, Fadare 2024 (a recent primary study on cold ischemia time in breast cancer biomarkers after neoadjuvant chemotherapy).

• References that were no longer cited in the revised text have been removed (Hammond 2010 superseded; Wolff 2013 and Wolff 2018 superseded; Goldstein 2003, Babawale 2014, Portier 2013, Bass 2014, Nkoy 2010, Gündisch 2012 — none of them cited in the revised text).

• All remaining references have been renumbered strictly in order of first appearance in the text, as required by PLOS ONE Vancouver style. The final reference list contains 25 entries, continuously numbered from 1 to 25.

We have verified that every in-text citation resolves to an existing reference, that every reference in the list is cited at least once in the text, and that reference numbers appear in strictly increasing order of first appearance in the text.

# **Response to Journal Requirements**

## **Requirement 1 — PLOS ONE style**

***Reviewer comment:*** *Please ensure that your manuscript meets PLOS ONE's style requirements, including those for file naming.*

**Author response:**

We have carefully reviewed the PLOS ONE formatting templates for both the title/authors/affiliations page and the main body. The revised manuscript has been reformatted accordingly: title page, author list and affiliations, section headings (Introduction, Materials and Methods, Results, Discussion, Conclusion), figure legends, reference style (Vancouver numerical with DOIs where available, numbered in order of first appearance), abbreviations, sentence-case title, and file naming conventions now conform to the PLOS ONE templates. Line numbers have been added throughout. Three separate files are uploaded as requested: (i) the unmarked revised manuscript, (ii) the revised manuscript with tracked changes, and (iii) this Response to Reviewers letter. A cover letter is also provided to summarize the key changes, including the revised title and the restructuring of the reference list.

## **Requirement 2 — Author and ethics committee**

***Reviewer comment:*** *We note that one of the authors of the present submission (Emile Telesphore MBOUDOU) is listed as a member of the ethics committee on the approval document provided. Could you please explain what your ethical committee's policies are when an Author is also a member of the committee, and how this was handled in this specific case?*

**Author response:**

Thank you for raising this important point. We would like to clarify that there was, in fact, no conflict of interest at the time of the ethics review of this research.

The ethics approval (N°2024/1617/HGOPED/DG/CEI) was issued by the Institutional Ethics Committee of the Douala Gynaeco-Obstetric and Pediatric Hospital (DGOPEH) for the doctoral (PhD) thesis of the first author, NDENGUE Clément Parfait, at the University of Douala. The thesis supervisors were Prof. ATANGANA Paul Jean Adrien and Prof. EBOUMBOU MOUKOKO Carole Else. At the time the research protocol was drafted, submitted to the committee, reviewed, and approved, Prof. MBOUDOU Emile Telesphore — who is Chair of the DGOPEH Institutional Ethics Committee — was not part of the research team, was not listed as an investigator on the protocol, and had no involvement whatsoever in the study design or in the submission. He may therefore have taken part in the committee's evaluation of the protocol in his institutional capacity as Chair, but without any conflict of interest, since he was not a co-investigator on the protocol being reviewed.

Prof. MBOUDOU was associated with the present work only later, during the preparation of the manuscript derived from the thesis. His contribution corresponds to the roles of Project administration and Resources, as listed in the Author Contributions section, and reflects his institutional position as General Director of DGOPEH at the time of manuscript preparation. In summary: he was a neutral evaluator at the time of ethics review (no conflict of interest) and only later joined the manuscript authorship.

We have added a brief clarifying statement to the Ethical Considerations subsection of the Materials and Methods so that readers of the published article can see the chronology transparently; the added statement is intentionally sober, mentions that Prof. MBOUDOU is Chair of the committee, and states the absence of conflict of interest at the time of protocol evaluation (rather than the absence of any involvement, which would be incorrect).

**Changes made to the manuscript:**

Materials and Methods — Ethical Considerations: added the sentence: "Prof. MBOUDOU Emile Telesphore, Chair of the DGOPEH Institutional Ethics Committee, was not part of the research team at the time of protocol submission and ethics review; he therefore had no conflict of interest with respect to this protocol during its evaluation by the committee. He was subsequently associated with the preparation of the present manuscript in his institutional capacity (Project administration, Resources)."

## **Requirement 3 — Citing specific previously published works**

***Reviewer comment:*** *If the reviewer comments include a recommendation to cite specific previously published works, please review and evaluate these publications to determine whether they are relevant and should be cited.*

**Author response:**

Reviewer #3 pointed us towards the foundational ASCO/CAP guideline evidence on cold ischemia time and breast biomarkers. We have evaluated these references for relevance and have expanded the Introduction and Discussion to cite the most current versions explicitly, replacing the superseded versions previously cited. The complete restructuring of the reference list is described above in the Preliminary Note. In brief, the revised list now includes:

• Allison KH et al. Estrogen and Progesterone Receptor Testing in Breast Cancer: ASCO/CAP Guideline Update. J Clin Oncol 2020 — primary guideline reference for ER/PR, replacing Hammond et al. 2010;

• Wolff AC et al. Human Epidermal Growth Factor Receptor 2 Testing in Breast Cancer: ASCO-CAP Guideline Update. J Clin Oncol 2023 — primary guideline reference for HER2, replacing Wolff 2013/2018;

• Shi WJ, Ghlichloo I, Fadare O. The effect of prolonged cold ischemia time on breast cancer biomarker expression after neoadjuvant chemotherapy. Ann Diagn Pathol 2024 — a recent primary study on cold ischemia time, cited in the Discussion alongside our cold ischemia findings;

• Khoury T. Delay to formalin fixation (cold ischemia time) effect on breast cancer molecules. Am J Clin Pathol 2018 — retained as the most comprehensive recent review.

Older foundational experimental studies (Khoury 2009, Yildiz-Aktas 2012, Li 2013, Neumeister 2012, Apple 2011, Oyama 2007, Kalkman 2014, Pinhel 2010, Fox 1985, Espina 2004, Viale 2007, Kiernan 2000) are retained where relevant to the revised discussion. Details of the revised Introduction and Discussion paragraphs are provided in our response to Reviewer #3, Minor Comment 1.

# **Response to the Academic Editor**

***Reviewer comment:*** *The primary concerns relate to the study design and interpretation of the results as pointed out by two reviewers. Overall, the study has value, but substantial revision is needed, particularly in framing, interpretation, and discussion of limitations.*

**Author response:**

We fully accept these concerns and have addressed them through systematic revision. In particular:

• Framing: the manuscript is now explicitly described — from the title onwards — as an exploratory, proof-of-concept, controlled experimental study on a single index tumor. Every section (abstract, introduction, methods, results, discussion, conclusion) has been rewritten to avoid any language implying population-level inference.

• Interpretation: we have separated statistical significance from biological and clinical relevance throughout the Results and Discussion. Small but statistically significant differences between fixatives are now reported as such, without being presented as evidence of equivalence.

• Limitations: the Limitations subsection has been restructured and expanded. The single-specimen design, the risk of pseudoreplication, the high baseline expression of the index tumor, intra-tumoral heterogeneity, and the limits of extrapolation to resource-limited clinical practice are now foregrounded rather than appearing near the end of the Discussion as caveats.

• Scope of the pre-analytical variables tested: the title, abstract, introduction, and discussion now consistently refer to all three variables investigated (fixation duration, cold ischemia time, and fixative preparation). The previously missing statistical analysis of the fixation-duration effect has been added, using the raw data provided in S1 Data. A new Discussion subsection dedicated to the Effect of Fixation Duration has also been added, so that each of the three variables is now discussed in its own dedicated subsection.

• References and supporting literature: the reference list has been entirely restructured to cite the current ASCO/CAP guidelines (Allison et al. 2020 for ER/PR; Wolff et al. 2023 for HER2) in place of their superseded predecessors, to include our own prior empirical observations at the same institution (Ndengue et al. 2025), and to add a recent 2024 primary study on cold ischemia time (Shi et al. 2024). All references have been renumbered in strict order of first appearance in the text, as required by PLOS ONE Vancouver style.

Detailed, point-by-point responses to the three reviewers follow.

# **Response to Reviewer #1**

***Reviewer comment:*** *This manuscript evaluates on the effect of cold ischemia time and fixative preparation on breast cancer biomarker expression. This manuscript is well-written and impactful for methods standardization in pathology workflows.*

**Author response:**

We thank Reviewer #1 for the positive evaluation and for recognizing the practical relevance of the study for pathology standardization. No specific change was requested; however, we note that the revisions performed in response to Reviewers #2 and #3 — notably the addition of statistical analysis of fixation-duration effects, the new Discussion subsection dedicated to fixation duration, the explicit acknowledgment of the single-specimen design, the grounding of the extended fixation range in our prior empirical work at the same institution, and the complete restructuring of the reference list — have further strengthened the manuscript in ways that we believe Reviewer #1 will find consistent with their overall assessment.

# **Response to Reviewer #2**

### ***Comment 1 — Overall assessment***

***Reviewer comment:*** *Good manuscript.*

**Author response:**

We thank the reviewer for this encouraging assessment.

### ***Comment 2 — Single specimen and variability between tissue sources***

***Reviewer comment:*** *Only one specimen has been used in this study although it has been divided into 50 sections. It might be useful to determine if there is variability between different tissue sources.*

**Author response:**

We agree entirely. The use of a single index specimen was a deliberate methodological choice: it allowed us to control inter-tumoral biological variability and attribute any observed differences exclusively to the three pre-analytical variables under study (fixation duration, cold ischemia time, and fixative preparation). This approach is consistent with the design used in several seminal pre-analytical studies (e.g., Khoury 2009 [8], Yildiz-Aktas et al. 2012 [11], Apple et al. 2011 [17]). However, as the reviewer correctly points out, this design cannot capture between-tumor biological variability, which is a fundamental limitation for clinical generalization.

We have addressed this comment in three complementary ways:

• We have re-framed the entire manuscript — including the title — as exploratory and proof-of-concept, explicitly stating that findings must be validated in multi-specimen studies before any practice recommendations are made;

• We have expanded the Limitations subsection with a dedicated paragraph on biological variability, distinguishing between intra-tumoral heterogeneity (addressed in part by sampling 50 microcores from different regions of the tumor) and inter-tumoral heterogeneity (which our design cannot address);

• We have added to the Future Research paragraph a specific call for multi-center, multi-specimen validation studies incorporating tumors with varying molecular subtypes, baseline expression levels, and histological grades.

**Changes made to the manuscript:**

Title: rewritten to include the wording "a controlled single-specimen study", making the design explicit from the first line.

Abstract — Conclusions: rewritten to begin with "In this single-specimen controlled experiment…" and to end with "…these findings require validation in multi-patient studies across diverse tumor subtypes."

Discussion — Limitations subsection: expanded with a new paragraph explicitly addressing the single-specimen design, the risk of pseudoreplication, and the distinction between intra- and inter-tumoral variability.

Discussion — Future Research paragraph: revised to include multi-specimen validation as the primary next step.

### ***Comment 3 — Missing statistical analysis of fixation duration***

***Reviewer comment:*** *Statistical significance for the fixation time is also not provided.*

**Author response:**

This is an important and correct observation. In the previous version, we reported Spearman correlations for cold ischemia time and a global two-way ANOVA for fixative × cold ischemia interaction, but we did not formally test the effect of fixation duration. We have now corrected this omission by conducting the following analyses on the raw data provided in S1 Data:

• Spearman rank correlation between fixation duration (0.5–144 h) and each biomarker, separately for each fixative;

• One-way ANOVA across four a priori fixation-duration windows (0.5–12 h, 24–48 h, 72–96 h, 120–144 h), separately for each fixative and each biomarker, followed by Welch pairwise t-tests between the reference window and the other windows.

The results confirm and quantify the trends previously described only verbally — biomarker expression is stable from 0.5 h to 48 h and then declines significantly at extended durations. The key values are:

Spearman correlations between fixation duration and percentage of positive cells:

• ER %: NBF ρ = −0.736, p = 0.0003; NB-F ρ = −0.658, p = 0.0022;

• PR %: NBF ρ = −0.738, p = 0.0003; NB-F ρ = −0.830, p < 0.0001;

• Ki67 %: NBF ρ = −0.596, p = 0.007; NB-F ρ = −0.646, p = 0.003.

One-way ANOVA across fixation-duration windows (all p < 0.05 for all biomarkers in both fixatives):

• ER %: NBF F = 22.96, p < 0.0001; NB-F F = 25.63, p < 0.0001;

• PR %: NBF F = 28.99, p < 0.0001; NB-F F = 45.48, p < 0.0001;

• Ki67 %: NBF F = 5.93, p = 0.007; NB-F F = 6.13, p = 0.006.

Pairwise comparisons (Welch t-test) between the 0.5–12 h reference window and the other windows show that the 24–48 h window is not significantly different from the reference (all p > 0.15), whereas the 120–144 h window differs significantly from the reference for ER and PR percentages in both fixatives. The stability of the biomarkers up to approximately 48 h and their significant decline beyond 72 h is therefore both visually evident in the data and statistically supported.

In addition, we have added a new Discussion subsection dedicated to the Effect of Fixation Duration (complementing the existing subsections on fixative preparation and cold ischemia time), so that the added statistical results are now properly interpreted alongside the relevant literature and the local empirical context (Ndengue et al. 2025).

**Changes made to the manuscript:**

Materials and Methods — Statistical Analysis: added description of Spearman correlations for fixation duration and one-way ANOVA across fixation windows, with Welch pairwise t-tests.

Results — Effect of Fixation Duration on Biomarker Expression: added quantitative statistical results (Spearman ρ, ANOVA F and p-values, pairwise comparisons).

Discussion: added new subsection "Effect of Fixation Duration" interpreting these findings in the context of prior literature and local practice.

S1 Data: statistical outputs for the added analyses included in the Statistical_Tests sheet.

# **Response to Reviewer #3**

We thank Reviewer #3 for a particularly thorough and constructive review. The comments have substantially shaped the revision. We address the major and minor comments in turn.

## **Major Comments**

### ***Major Comment 1 — Single-patient design and pseudoreplication***

***Reviewer comment:*** *The study is based on tissue from a single patient, with multiple microsamples treated as experimental units. While this may be acceptable for a pilot or proof-of-concept study, it raises concerns regarding biological independence and pseudoreplication, particularly given the use of statistical testing. The study should be explicitly framed as exploratory, and language implying population-level inference should be avoided.*

**Author response:**

We fully accept this criticism. Our original framing did not sufficiently acknowledge that the 50 microcores, although fixed and processed independently, derive from a single biological donor and therefore do not provide independent estimates of the true population-level effect of fixation duration, cold ischemia time, or fixative preparation. The correlations and p-values we report quantify within-specimen technical variability under controlled pre-analytical conditions; they do not license population-level inference.

The revised manuscript has been systematically re-framed as follows:

• The title itself now reads "…a controlled single-specimen study of fixation duration, cold ischemia time, and fixative preparation in a low-resource setting", making the design explicit from the first line;

• The Abstract now opens its Conclusions with "In this single-specimen controlled experiment…" and closes with a call for multi-patient validation;

• The Introduction's final paragraph now ends with: "We present this work as an exploratory, proof-of-concept experimental study intended to generate hypotheses for subsequent multi-specimen validation";

• The Statistical Analysis subsection now explicitly states that p-values describe within-specimen technical variation and do not support population-level inference;

• Throughout the Results and Discussion, language such as "preserves", "demonstrates", and "confirms" has been replaced with "was associated with", "was consistent with", or "suggests, in this single specimen";

• The Conclusion has been substantially toned down (see our response to Major Comment 5).

**Changes made to the manuscript:**

Changes appear throughout the manuscript and are visible in the tracked-changes version. Key locations: Title and Running Title; Abstract — Conclusions; Introduction — final paragraph; Materials and Methods — Statistical Analysis; Discussion — Limitations; Conclusion.

### ***Major Comment 2 — Intra-tumoral ER heterogeneity and high baseline expression***

***Reviewer comment:*** *Estrogen receptor expression is known to show spatial heterogeneity within invasive breast carcinomas, as well as variability across different areas of the breast tissue more broadly. Sampling multiple microspecimens from a single tumour does not fully account for this biological variability and may bias results toward regions with uniformly high ER expression. This limitation is particularly relevant given the high baseline ER positivity in the index case, where small fixation-related changes are unlikely to affect categorical scoring. The authors should explicitly acknowledge that heterogeneous or low-ER tumours may be more vulnerable to pre-analytical variation, and that findings from this single-tumour model may not extrapolate to the full spectrum of ER expression patterns seen in clinical practice. This could also be true for Ki67.*

**Author response:**

This is an excellent and fundamental point, which we have addressed substantively in the revision. Our index tumor was strongly ER- and PR-positive (approximately 100% and 95–100% positive cells, respectively) and highly proliferative (Ki67 = 40%). Under these conditions, small pre-analytical perturbations are unlikely to shift categorical classification (positive vs. negative; high- vs. low-proliferative) because the specimen sits far from the clinical decision thresholds. Tumors that sit close to diagnostic cut-offs — weakly ER-positive tumors (ER-low-positive, 1–10% positive cells, as defined in the ASCO/CAP 2020 update [4]), heterogeneously stained tumors, or tumors with Ki67 near the 10% and 30% boundaries — are the ones most likely to be reclassified by pre-analytical variation. Our data cannot speak to those cases, and we have made this explicit.

We have added a new paragraph to the Limitations subsection dedicated to this point, with the following wording:

*"The index tumor exhibited strong, diffuse ER and PR positivity and high Ki67, placing it far from the diagnostic decision thresholds (1% for hormone-receptor positivity, with the 1–10% "ER-low-positive" range now defined in the ASCO/CAP 2020 update [4]; 10% and 30% for Ki67 proliferation categories). In this setting, even biologically meaningful declines in staining are unlikely to shift categorical classification. Tumors with lower or heterogeneous baseline expression — particularly those with weak (1–10%) ER or PR positivity, focal or mosaic staining patterns, or Ki67 near the 10–30% range — are intrinsically closer to these thresholds and may therefore be reclassified by the same absolute magnitude of pre-analytical loss observed here. Intra-tumoral heterogeneity of ER, PR, and Ki67 is well documented in invasive breast carcinoma and was not systematically addressed by our sampling strategy; the 50 microcores were drawn from different regions of the tumor mass but may nevertheless have been enriched for regions with uniformly strong expression. Our findings should therefore not be extrapolated to the full spectrum of ER, PR, or Ki67 expression patterns encountered in clinical practice."*

We have also added a cautionary sentence to the Abstract Conclusions and to the Conclusion section.

**Changes made to the manuscript:**

Discussion — Limitations: new dedicated paragraph on intra-tumoral heterogeneity and baseline-expression dependence of pre-analytical vulnerability (quoted above).

Abstract — Conclusions: added "…and warrant further investigation across diverse tumor types and baseline expression levels, particularly tumors with ER-low-positive (1–10%) or heterogeneous expression."

Conclusion (main text): added explicit statement that findings may not extend to tumors with low or heterogeneous baseline biomarker expression.

### ***Major Comment 3 — Limited novelty relative to existing literature***

***Reviewer comment:*** *Previous studies have independently examined the impact of cold ischemia time on breast cancer biomarkers and buffered versus unbuffered formalin fixation for ER and Ki67 evaluation. The principal novelty appears to lie in the combined, controlled evaluation of fixation delay and fixative preparation. This distinction should be stated more clearly to avoid the impression that the individual findings are novel.*

**Author response:**

We agree. The individual effects of fixation duration, cold ischemia time, and fixative preparation on breast biomarkers have each been studied independently in prior work. What the existing literature does not provide, to our knowledge, is a single controlled experiment in which these three pre-analytical variables are manipulated in a factorial design on material from the same tumor, with a specific focus on the formaldehyde preparations actually in use in sub-Saharan African pathology laboratories (in-house diluted non-buffered formaldehyde versus commercial 4% NBF). We have rewritten the relevant paragraph in the Introduction to make this distinction explicit.

The revised paragraph reads:

*"Previous studies have separately examined the effect of prolonged fixation duration on hormone-receptor and HER2 expression [17–20], the effect of cold ischemia time on the same biomarkers [8,11–14,21], and the effect of different formalin preparations on immunohistochemical assessment of ER, PR, and Ki67 [17,18]. To our knowledge, however, no single controlled experiment has simultaneously varied all three of these pre-analytical variables — fixative preparation (4% neutral buffered versus 4% non-buffered formaldehyde prepared in-house), fixation duration (0.5 h to 144 h), and cold ischemia time (0.5 h to 8 h) — on material from the same invasive breast carcinoma, with a design specifically motivated by the fixative preparations actually available in sub-Saharan African pathology laboratories. The novelty of the present study therefore lies not in any of the individual factors, which are confirmatory, but in their combined, controlled, factorial evaluation on a single tumor in a setting representative of low-resource pathology practice."*

**Changes made to the manuscript:**

Introduction — penultimate paragraph rewritten as quoted above, explicitly positioning the combined factorial design as the locus of novelty and acknowledging that the individual findings are confirmatory.

Discussion — Principal Findings: added a sentence clarifying that the cold-ischemia-time and fixative-type findings individually replicate prior work, and that the contribution of the present study is in their joint evaluation.

### ***Major Comment 4 — Statistically significant but small differences; relevance of extended fixation times***

***Reviewer comment:*** *The manuscript reports statistically significant differences in ER and PR positivity percentages between fixatives, yet Allred scores remain unchanged. This raises questions regarding biological and clinical relevance. The conclusion that non-buffered formaldehyde preserves biomarkers "comparably" should be tempered to acknowledge that measurable differences, albeit small, were observed. Why include fixation times of up to 144 hours as they exceed typical diagnostic workflows. The clinical relevance of these extremes should be explained or contextualised.*

**Author response:**

We agree on both points and have addressed them separately.

**(a) Interpretation of small but statistically significant differences.** The revised Discussion now distinguishes clearly between statistical and biological or clinical relevance. We no longer claim that non-buffered formaldehyde preserves biomarkers "comparably" to NBF. We instead state that NBF was associated with small but statistically significant increases in mean percentage of positive cells and staining intensity for ER and PR (ΔER = 2.58%, ΔPR = 2.26%, ΔER intensity = 0.18, ΔPR intensity = 0.21), and that in this index tumor these differences did not translate into categorical reclassification by the Allred system. We explicitly caution that in tumors with weaker baseline expression, the same absolute differences could cross the Allred threshold.

**(b) Inclusion of fixation times up to 144 h.** The reviewer is correct that 144 h exceeds the ASCO/CAP-recommended 6–72 h window for routine diagnostic fixation. This choice was, however, not speculative but empirically grounded in our local practice. In a prior retrospective analysis from the same laboratory (Ndengue et al., J Sci Dis 2025;3(2):68–74 [22]), we documented that 85.9% of invasive breast cancer specimens processed at the DGOPEH pathology laboratory between 2017 and 2020 underwent prolonged fixation. Fixation durations varied by specimen type: biopsies reached 18–83 h, nodulectomies 19–82 h, and mastectomy specimens 43–147 h (mean 88.3 ± 5.4 h for mastectomies). Our 14-gauge microcores are morphologically analogous to biopsies, but the 0.5–144 h range tested in the present study was deliberately chosen to span the full distribution of fixation durations observed in routine practice across all specimen types at our institution, thereby providing technical benchmarks applicable to biopsies, nodulectomies, and mastectomies alike. Durations of 72–144 h correspond to specimens held in fixative over weekends or public holidays, a recurring logistical reality in resource-limited settings with limited pathology laboratory staffing. We have added this rationale to the Materials and Methods and to the Discussion, while maintaining the caveat that fixation durations beyond 72 h lie outside the ASCO/CAP-recommended window and should be interpreted as technical benchmarks rather than endorsed clinical practice.

**Changes made to the manuscript:**

Discussion — Principal Findings and Fixative Preparation: rewritten to replace "comparably" with language that acknowledges the measured differences and warns about tumors near the diagnostic threshold.

Materials and Methods — Experimental Groups: added rationale for the 0.5–144 h fixation range, with citation of Ndengue et al. 2025 [22] documenting fixation-duration distributions by specimen type (biopsies, nodulectomies, mastectomies).

Introduction — final paragraph: added citation of Ndengue et al. 2025 [22] to motivate the experimental study with empirical local data, including the biopsy/nodulectomy/mastectomy distinction.

Discussion — new subsection "Effect of Fixation Duration": provides interpretation of the fixation-duration findings, including the clinical relevance of extended durations and their empirical observation in local practice.

Discussion — added a sentence clarifying that 72–144 h fixation durations are outside the ASCO/CAP-recommended window and are presented as technical benchmarks relevant to observed local practice.

Reference list: Ndengue CP et al. Effect of fixation time and sample type on tissue biomarker preservation in breast cancer in Cameroon. J Sci Dis 2025;3(2):68–74 (reference [22] in the renumbered list).

### ***Major Comment 5 — Overgeneralization to resource-limited settings***

***Reviewer comment:*** *While the study is motivated by challenges faced by laboratories in low-resource settings, extrapolating from a highly controlled, single-tumour experiment to real-world practice may be premature. Although the need for validation is noted, this limitation should be more prominently foregrounded.*

**Author response:**

We agree. The original manuscript acknowledged this limitation, but the caveat was placed in the latter part of the Discussion and was not prominent enough relative to the practical recommendations. We have restructured the Discussion and Conclusion so that the limits of extrapolation to real-world resource-limited practice are foregrounded rather than buried.

Specifically:

• The title itself now ends with "…in a low-resource setting" (singular), reflecting the single-centre, single-specimen nature of the work;

• The first sentence of the Discussion now explicitly states that the findings are exploratory and derived from a single, highly controlled experiment;

• The Practical Implications subsection has been rewritten to use conditional language ("these findings suggest, if confirmed in multi-specimen studies, that…") rather than direct recommendations;

• The Conclusion now opens by stating the exploratory nature of the work and ends by emphasizing that multi-center validation is required before any practice change is considered;

• The practical workflow suggestions (fixative containers in operating rooms, staff training, cold-ischemia-time documentation, etc.) are retained but are now clearly framed as generic best-practice recommendations already supported by existing ASCO/CAP guidelines, and not as novel recommendations arising from our data.

**Changes made to the manuscript:**

Title: changed from "Implications for Resource-Limited Settings" to "in a low-resource setting" (singular).

Discussion — first paragraph rewritten to foreground the exploratory, single-specimen nature of the study.

Discussion — Practical Implications and Study Limitations: subsection restructured so that limitations precede implications, and implications are expressed in conditional form.

Conclusion: substantially revised — first and last sentences now state the exploratory nature and the need for multi-center validation; the intermediate practical-workflow suggestions are clearly framed as already-established best practice.

## **Minor Comments**

### ***Minor Comment 1 — Missing key breast-specific literature***

***Reviewer comment:*** *Multiple prior studies and ASCO/CAP guideline evidence demonstrate that ER, PR, and HER2 expression declines with prolonged cold ischemia, with ≤1 hour considered optimal and deterioration becoming detectable beyond 1–2 hours. These foundational studies should be explicitly acknowledged.*

**Author response:**

We agree and have expanded our coverage of the foundational literature. In the previous version, several of these references were cited but were not explicitly connected to the ≤1 h optimal / 1–2 h deterioration-threshold evidence, and the cited guidelines were in their older (now superseded) versions. We have revised the Introduction and the Cold Ischemia Time subsection of the Discussion to cite the most current guidelines explicitly, including:

• Allison KH et al. 2020 ASCO/CAP ER/PR guideline update [ref 4] (replaces Hammond et al. 2010 as the current ER/PR guideline);

• Wolff AC et al. 2023 ASCO-CAP HER2 guideline update [ref 5] (replaces Wolff 2013 and Wolff 2018 as the current HER2 guideline);

• Khoury T. 2018 comprehensive review of delay to formalin fixation [ref 12] (retained as the most comprehensive recent review);

• Shi WJ, Ghlichloo I, Fadare O. 2024 primary study on prolonged cold ischemia after neoadjuvant chemotherapy [ref 21] (newly added, cited in the Discussion);

• Khoury T et al. 2009 [ref 8]; Yildiz-Aktas et al. 2012 [ref 11]; Li et al. 2013 [ref 13]; Neumeister et al. 2012 [ref 14] — retained as the foundational experimental evidence on cold ischemia effects.

The revised Introduction now contains an explicit sentence: "Prior foundational work has established that cold ischemia times ≤1 hour are optimal for ER, PR, and HER2 preservation, with detectable deterioration beyond 1–2 hours and progressive loss thereafter [8,11–14]." The corresponding passage in the Discussion has been expanded to place our findings in the context of this prior evidence, and the Shi et al. 2024 study is now cited in the Critical Impact of Cold Ischemia Time subsection.

**Changes made to the manuscript:**

Introduction — second paragraph: explicit statement of the ≤1 h / 1–2 h threshold evidence with current guideline citations.

Discussion — Critical Impact of Cold Ischemia Time: expanded to connect our Spearman correlations and 2-h threshold to the current ASCO/CAP guidelines and foundational experimental evidence, with added citation of Shi et al. 2024.

Reference list: fully restructured and renumbered in order of first appearance; includes Allison 2020, Wolff 2023, Shi et al. 2024, Ndengue 2025; superseded guidelines and uncited references have been removed.

### ***Minor Comment 2 — Clarity of sample description***

***Reviewer comment:*** *The reported microsample dimensions ("5–15 mm in length, 130 mm in width") appear unusual and may reflect a typographical or unit error.*

**Author response:**

Thank you for this observation. We note that the manuscript as submitted consistently describes the microcores as "5–15 mm in length and 1 mm in diameter" (in the Materials and Methods) and "5–15 mm in length, 1 mm in width" (in the Abstract), which are consistent with 14-gauge core needle biopsy sampling. The figure "130 mm" mentioned by the reviewer does not appear in our submitted manuscript; we suspect it corresponds to an on-screen misreading of "1 mm". Nevertheless, to eliminate any possibility of ambiguity, we have harmonized the wording between Abstract and Materials and Methods, and we have added the biopsy device specification.

Revised wording (Materials and Methods): "Fifty microsamples were obtained from the tumor mass using a 14-gauge core needle biopsy device. Each microcore measured approximately 5–15 mm in length and approximately 1 mm in diameter."

The Abstract has been updated to match: "Fifty microsamples (5–15 mm in length, approximately 1 mm in diameter) were obtained…"

**Changes made to the manuscript:**

Abstract — Methods: updated to "Fifty microsamples (5–15 mm in length, approximately 1 mm in diameter) were obtained…"

Materials and Methods — Samples Collection: updated as quoted above, with explicit mention of the 14-gauge core needle biopsy device.

# **Closing**

We thank the editor and the three reviewers once again for their thoughtful and constructive input. We believe the revised manuscript is substantially strengthened, with a clearer framing as exploratory work from the title onwards, a more rigorous treatment of statistical significance in relation to clinical relevance, a more honest account of the study's limitations, a more faithful positioning within the existing literature on breast cancer pre-analytics, the grounding of our extended fixation range in our own prior empirical observations at the same institution (with explicit distinction between biopsies and mastectomies), a new Discussion subsection dedicated to fixation duration, and a fully restructured and renumbered reference list conforming to PLOS ONE Vancouver style. We hope the revised version meets the standards of PLOS ONE and look forward to your decision.

Yours sincerely,

NDENGUE Clément Parfait, on behalf of all co-authors

Corresponding author

Douala Gynaeco-Obstetric and Pediatric Hospital, Douala, Cameroon

E-mail: ndengueparfait@yahoo.fr
