# Supplementary material for: Pre-analytical variables affecting breast cancer biomarker expression: A controlled single-specimen study of fixation duration, cold ischemia time, and fixative preparation in a low-resource setting
Source: PLoS One. 2026 Jun 5;21(6):e0343185. doi: 10.1371/journal.pone.0343185 (PMC13240924; doi:10.1371/journal.pone.0343185)
Supplement: Attachment — Submitted filename: Response_to_Reviewers_PONE-D-26-05253_R2v2.docx [file pone.0343185.s004.docx]

**Response to Reviewers (Second Revision)**

**Manuscript PONE-D-26-05253R1**

*Pre-analytical variables affecting breast cancer biomarker expression: a controlled single-specimen study of fixation duration, cold ischemia time, and fixative preparation in a low-resource setting*

Dear Dr. Kaminski,

We sincerely thank you and the reviewers for the constructive evaluation of the first revision of our manuscript. We are gratified that Reviewer #2 and Reviewer #3 have indicated that all of their previous comments have been adequately addressed, and that the manuscript is considered technically sound, with appropriate statistical analyses and complete data availability.

In your editorial letter, you raised two final minor points before the manuscript can proceed toward acceptance:

• (a) Provide additional antibody information, including catalog numbers and concentrations/dilutions used.

• (b) Include a supplementary figure showing representative staining images observed in the study.

We address both points below, with full transparency. We are pleased to fully resolve point (a) and we present a candid account of point (b), together with a request for the editor's guidance on the best path forward should representative images be considered essential for publication.

# Point (a) — Additional antibody information

***Editor / Reviewer comment:*** *Please provide additional antibody information, including catalog numbers and concentrations/dilutions used. (Editor; also raised by Reviewer #3.)*

**Author response:**

We agree entirely and have fully addressed this point. The Tissue Processing and Immunohistochemistry subsection of the Materials and Methods has been substantially expanded to include manufacturer details (Ventana Medical Systems, Roche Diagnostics, Tucson, AZ, USA), catalog numbers for each primary antibody (across both available pack sizes, since the small-volume and large-volume formats of the CONFIRM range share the same antibody clone and manufacturer-optimized concentration), the manufacturer-optimized working concentration (~1 µg/mL, ready-to-use format), and the catalog numbers of the ancillary reagents used in the protocol (cell-conditioning solution, detection kit, counterstain, and negative-control reagent).

The three primary antibodies used in the study are now reported as:

• CONFIRM anti-Estrogen Receptor (ER) clone SP1 (Roche Diagnostics, Cat. Nos. 790-4324 [50 tests] or 790-4325 [250 tests]); rabbit monoclonal, ready-to-use, ~1 µg/mL.

• CONFIRM anti-Progesterone Receptor (PR) clone 1E2 (Roche Diagnostics, Cat. Nos. 790-2223 [50 tests] or 790-4296 [250 tests]); rabbit monoclonal, ready-to-use, ~1 µg/mL.

• CONFIRM anti-Ki-67 clone 30-9 (Roche Diagnostics, Cat. No. 790-4286, 50 tests); rabbit monoclonal, ready-to-use.

The ancillary reagents are now also specified by catalog number:

• Cell Conditioning Solution 1 (CC1, pH 8.0; Cat. No. 950-124) for heat-induced epitope retrieval.

• OptiView DAB IHC Detection Kit (Cat. No. 760-700) for chromogenic visualization.

• Hematoxylin II (Cat. No. 790-2208) for counterstaining.

• CONFIRM Negative Control Rabbit Ig (Cat. No. 760-1029) included as negative reagent control in each immunostaining run, in addition to positive and negative tissue controls.

**Changes made to the manuscript:**

Materials and Methods — Tissue Processing and Immunohistochemistry: the antibody and reagent paragraph has been rewritten to incorporate the catalog numbers, clones, manufacturer details, and the ready-to-use concentration of approximately 1 µg/mL recommended by the manufacturer. The expanded text appears in blue in the tracked-changes version of the manuscript.

# Point (b) — Supplementary figure with representative staining images

***Editor / Reviewer comment:*** *Please include a supplementary figure showing representative staining observed in the study. (Editor; also raised by Reviewer #3.)*

**Author response:**

We thank the editor and Reviewer #3 for this constructive suggestion, which we have considered carefully. Regrettably, after due investigation, we are unable to provide a supplementary figure of representative immunohistochemistry images for the present revision. Below, we provide a full and transparent account of the circumstances and of the methodological elements that, in our view, mitigate the absence of representative images.

## Why representative images cannot be provided

The immunohistochemical staining of the 50 microcores for ER, PR, and Ki67 was performed in 2023 at a commercial pathology laboratory operating an automated BenchMark ULTRA platform (Ventana / Roche Diagnostics). The use of a fee-for-service commercial provider is a common arrangement in sub-Saharan African pathology practice, where in-house automated immunohistochemistry is not consistently available and where research projects routinely outsource the staining step to a commercial laboratory. The original stained slides were not archived by the commercial provider beyond their routine retention period, and despite our efforts to retrieve them in the context of the present revision, they are no longer accessible.

Digital images of the stained slides were not captured at the time of original assessment, because the study design relied on direct microscopic scoring by two independent board-certified observers using standardized criteria, with all quantitative results (percentages of positive cells, staining-intensity scores, Allred scores) recorded numerically for each of the 50 microcores. In retrospect, we recognize that systematic digital image capture should have been incorporated into the study protocol, and we have added a corresponding recommendation to the Discussion (Limitations subsection) so that future studies of pre-analytical variation in resource-limited settings will benefit from this lesson.

We note that the paraffin tissue blocks corresponding to the 50 microcores are conserved at the Anatomic Pathology Department of the Douala Gynaeco-Obstetric and Pediatric Hospital (DGOPEH). It would therefore be theoretically possible to recut the blocks and reproduce the immunostaining at a commercial laboratory. However, given that the present study was carried out as part of a self-funded doctoral thesis without external research funding, and given the cost structure of commercial fee-for-service immunohistochemistry (where each new run involves recutting, deparaffinization, antigen retrieval, three primary-antibody assays, and detection), reproducing even a small representative subset of the original staining would impose a substantial financial burden that the corresponding author would have difficulty meeting within the revision window.

## Methodological elements that support the validity of the immunohistochemical data

We respectfully submit that the rigor of the immunohistochemical assessment described in the manuscript is supported by several methodological elements that compensate, in part, for the absence of representative images:

• Staining was performed on a manufacturer-standardized automated platform (BenchMark ULTRA, Ventana / Roche Diagnostics) using manufacturer-validated ready-to-use rabbit monoclonal antibodies (CONFIRM range) at the optimized working concentration of ~1 µg/mL, minimizing technical variability associated with manual staining and antibody dilution.

• Each immunostaining run included appropriate positive and negative tissue controls (per the manufacturer's recommendation) together with a CONFIRM Negative Control Rabbit Ig reagent control, with the run validated only when these controls produced the expected staining patterns.

• All 50 microcores were scored independently by two board-certified observers blinded to experimental conditions, with discrepancies resolved by averaging.

• Inter-observer agreement was excellent: intraclass correlation coefficient = 0.94 for estrogen receptor, 0.92 for progesterone receptor, and 0.96 for Ki-67, all consistent with the upper range of agreement reported in published pre-analytical immunohistochemistry studies.

• The complete raw scoring data for each of the 50 microcores (proportion scores, intensity scores, Allred scores, and Ki-67 percentages) are provided in the S1 Data file, allowing every descriptive and inferential statistic in the manuscript to be independently reproduced from the raw data.

## Path forward and request for editorial guidance

We have added an explicit statement to the Limitations subsection of the Discussion in which we acknowledge the absence of representative microscopic images, describe the reason transparently, and recommend that future studies in this domain incorporate systematic digital image capture. This statement appears in blue in the tracked-changes version of the manuscript.

We respectfully request the editor's guidance on whether this explicit acknowledgment, in conjunction with the enriched antibody information now provided in the Methods section and the complete quantitative data available in S1 Data, can be considered sufficient for publication. Should the editor judge that representative images remain indispensable, we would welcome the opportunity to discuss alternative options, which might include (i) a substantial extension of the revision deadline so that we may attempt to identify funding or institutional support to recut and re-stain a representative subset of the paraffin blocks at a commercial laboratory, or (ii) any other path that the editorial team would consider appropriate. We remain fully committed to the scientific integrity of the work and to the journal's standards of methodological transparency, and we will follow the editor's decision on this point.

**Changes made to the manuscript:**

Discussion — Limitations subsection: a new paragraph has been added at the end of the limitations subsection to acknowledge the absence of representative microscopic images, to provide a transparent account of the underlying circumstances, and to recommend systematic image capture for future studies. The new paragraph appears in blue in the tracked-changes version of the manuscript.

# Additional corrections detected during the final pre-submission check (R2 v2)

While preparing the present resubmission, we identified three small items during a final pre-submission technical check that we have taken the opportunity to correct. None of these affect any data, analysis, or conclusion in the manuscript.

**(i) Author Contributions.** We have removed the line “Funding acquisition: EBOUMBOU MOUKOKO Carole Else” from the Author Contributions section, as this role was inconsistent with the Funding Statement of the manuscript, which declares that no specific funding was received for this work. EBOUMBOU MOUKOKO Carole Else remains credited for Supervision; only the Funding acquisition role has been removed.

**(ii) Figure Legends.** We have reworded the legends of Figures 1, 2, and 3 to accurately describe the panel layout actually shown in the TIFF files. The previous wording referred to sub-panels (A-B, C-D, E-F), implying that each biomarker was presented in two separate panels (one per fixative), whereas the figures in fact present a single panel per biomarker with the NBF (blue) and NB-F (magenta) curves overlaid. The Figure 3 legend has additionally been corrected to remove a sentence referring to a “bottom panel” showing staining-intensity decline, which does not exist in the figure.

**(iii) Figure 3 (Fig3.tiff) and corresponding S1 Data cleanup.** We have replaced the Figure 3 TIFF file to remove two annotations inadvertently retained in the previous export: a placeholder caption (“Data to be added from Cohorts 3 & 4 (delayed fixation experiments)”) that overlapped the x-axis at the lower left of the figure, and two orphan labels (“High” and “Low”) in the right margin of the Ki67 panel. The replacement Figure 3 has been regenerated from the raw Cold_Ischemia_Data values in the S1 Data file, with no change to the underlying data values; the High (>30%) and Low (<10%) Ki67 classification thresholds are now properly indicated by horizontal dashed lines with appropriately positioned labels inside the plot area. A corresponding minor cleanup has also been applied to the S1 Data file (Cold_Ischemia_Data sheet): the same residual placeholder text row that had been inadvertently retained from an earlier draft of the dataset has been removed. All numerical values in S1 Data are unchanged; a revision note documenting this cleanup has been added to the Metadata sheet of S1 Data.

**Changes made to the manuscript and to the supporting files:**

Manuscript — Author Contributions: the “Funding acquisition” line has been removed (shown in red strikethrough in the tracked-changes version of the manuscript). Manuscript — Figure Legends: the bodies of the Fig 1, Fig 2, and Fig 3 legends have been rewritten (deletions in red strikethrough, new wording in blue in the tracked-changes version). Figure 3 TIFF file: replaced with a corrected version regenerated from raw S1 Data values. S1 Data file: the placeholder row in the Cold_Ischemia_Data sheet has been removed and a revision note added to the Metadata sheet.

# Closing

We are grateful to you and to the two reviewers for the rigor and constructive spirit of the entire review process. We believe that the present revision fully resolves the antibody-information point and provides a transparent and complete account of the constraints affecting the supplementary-figure point. We very much hope that the manuscript can now proceed toward acceptance, and we remain at your disposal for any further clarification or adjustment.

Yours sincerely,

Dr NDENGUE Clément Parfait, on behalf of all co-authors

Corresponding author

Douala Gynaeco-Obstetric and Pediatric Hospital, Douala, Cameroon

E-mail: ndengueparfait@yahoo.fr
